# 618. Characteristics and Outcomes of an Outpatient Infectious Diseases E-consult Program at a County Safety-Net Healthcare System

**DOI:** 10.1093/ofid/ofab466.816

**Published:** 2021-12-04

**Authors:** Madison Granger, Madison Pickering, Richard J Medford, Helen King

**Affiliations:** 1 UT Southwestern Medical School, Dallas, TX; 2 UT Southwestern Medical Center, Richardson, TX; 3 University of Texas Southwestern, Dallas, TX

## Abstract

**Background:**

Safety-net healthcare systems often have significant demands for specialty care due to large patient volumes. Infectious Disease (ID) e-consults have the capability to relieve some of this burden by presenting providers with an alternative to face-to-face ID referrals that also lessens financial, travel, and time constraints on patients. Such a system offers the prospect of increasing access to ID care for patients in limited resource settings.

**Methods:**

We performed a retrospective review describing characteristics and outcomes of all outpatient ID e-consults at Parkland Health and Hospital System in Dallas, Texas from March 2018 – February 2021.

**Results:**

In the study period, 725 e-consults were completed. All e-consults were answered within 72 hours per hospital policy. The most common e-consult topics were 135 (19%) tuberculosis (TB), 116 (16%) syphilis, 97 (13%) respiratory and 79 (11%) musculoskeletal (Figure 1). Nearly two-thirds of the e-consults 456 (63%) came from primary care providers (PCPs). The remainder came from specialists with the most common referring specialties being GI 55 (8%), Hematology/Oncology 36 (5%), Rheumatology 28 (4%) Neurology 27 (4%), and Dermatology 22 (3%) (Figure 2). The majority of e-consults 569 (78%) were resolved without a face-to-face visit.

Figure 1. Number of E-consults over Time, by Topic

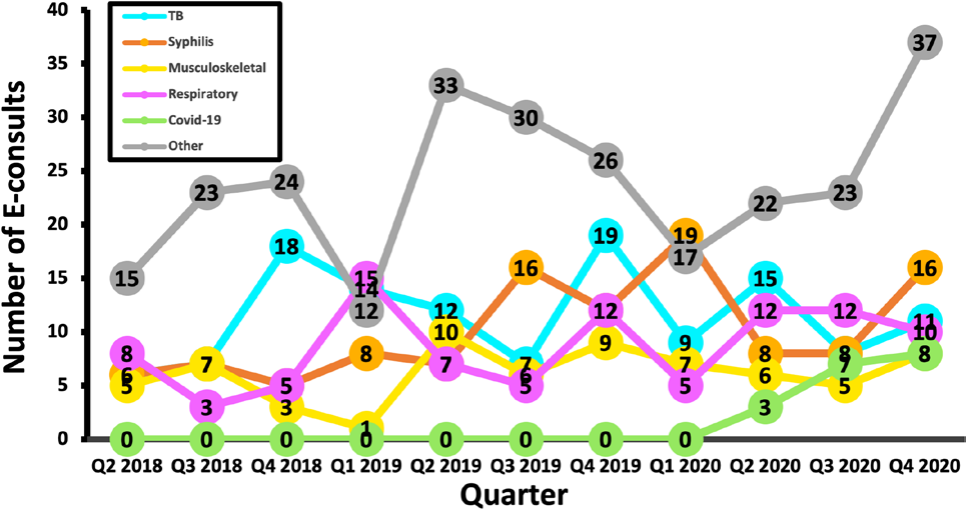

Figure 2. E-consult Topics by Referring Specialty

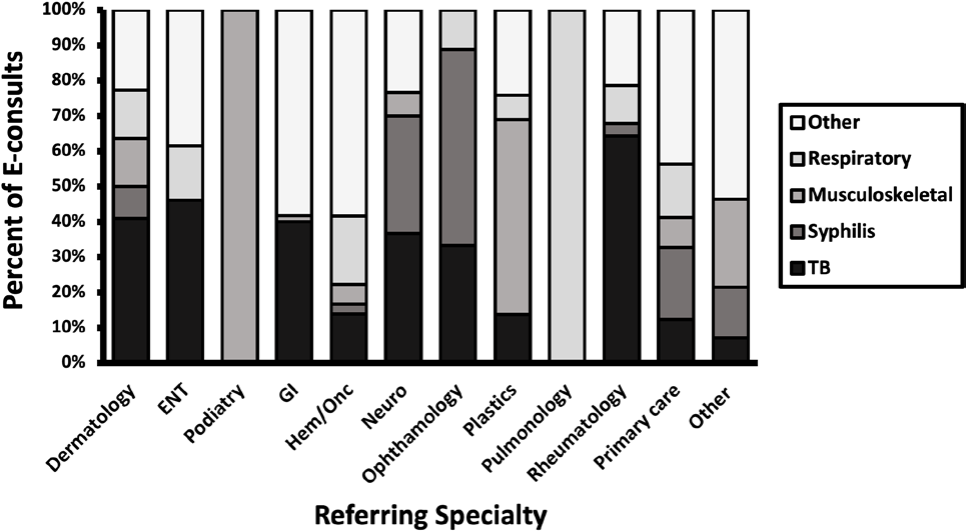

**Conclusion:**

Implementation of an outpatient ID e-consult program at a large safety-net healthcare system was an effective means of providing timely input on common ID topics, such as latent TB and interpretation of syphilis serologies, without formal clinic visits. E-consults were able to service a range of providers including PCPs and a variety of specialties, and most e-consults were completed without a clinic visit.

**Disclosures:**

**All Authors**: No reported disclosures

